# Well-being among parents of youth with multiple sclerosis: a preliminary longitudinal study

**DOI:** 10.3389/fpsyg.2024.1308141

**Published:** 2024-01-31

**Authors:** Liat Hamama, Yaira Hamama-Raz, Keshet Lebowitz-Sokolover, Esther Ganelin-Cohen

**Affiliations:** ^1^Bob Shapell School of Social Work, Tel Aviv University, Tel Aviv, Israel; ^2^School of Social Work, Ariel University, Ariel, Israel; ^3^Social work department, Schneider Children Medical Center, Petach Tikva, Israel; ^4^Institute of Pediatric Neurology, Schneider Children’s Medical Center, Petach Tikva, Israel; ^5^Sackler School of Medicine, Tel-Aviv University, Tel-Aviv, Israel

**Keywords:** pediatric-onset multiple sclerosis, parents, perceived social support, coping flexibility, satisfaction with life, psychological distress

## Abstract

**Background:**

In 2021, the annual rate of pediatric-onset multiple sclerosis (POMS) in Israel among children was 1.5, and 4.5% among youth aged 14–18, out of a total of 5,000 multiple sclerosis cases nationwide. Children diagnosed with POMS often display various deficiencies across psychological, cognitive, sensory, and physical areas. As such, POMS poses significant challenges for parents’ well-being, with heightened emotional, financial, and physical strains linked to both the immediate and long-term care requirements of their children. In this preliminary study, we examined changes over three time-points in two measures of well-being: satisfaction with life and psychological distress. In addition, the role of perceived social support (PSS) and coping flexibility was examined through a multilevel causal mediation model which suggested that PSS 1 month post-diagnosis would predict coping flexibility at 6 months post-diagnosis, which in turn would predict parents’ life satisfaction and psychological distress at 12 months post-diagnosis.

**Methods:**

The research was conducted at a tertiary university-affiliated children’s hospital in central Israel. Preliminary data were obtained from 36 parents at three times-points. Participants provided demographic information and filled out the following standardized self-report questionnaires: the Diener’s Satisfaction with Life Scale, Kessler’s inventory for measuring psychological distress (K6), the Multidimensional Scale of Perceived Social Support (MSPSS), and the Perceived Ability to Cope with Trauma Scale (PACT) for measuring coping flexibility.

**Results:**

Over time (12 months), parents reported stable levels of PSS, coping flexibility, satisfaction with life, and psychological distress. In addition, mothers reported significantly greater PSS from friends than did fathers. Regarding the causal mediation model, greater PSS from friends at T1 was significantly associated with an increase in coping flexibility from T1 to T2. In turn, an increase in coping flexibility was associated with a decrease in psychological distress from T1 to T3 (after controlling for PSS). Yet the causal mediation path via coping flexibility to satisfaction with life was not significant.

**Conclusion:**

This preliminary study emphasizes the important role of both PSS and coping flexibility for the well-being of parents whose children are affected by POMS, a subject that merits heightened consideration among healthcare professionals dealing with long-term chronic diseases.

## Introduction

Pediatric-onset multiple sclerosis (POMS) is a long-term condition that impacts the central nervous system in individuals under 18 years of age, leading to both cognitive and physical impairments (Portaccio et al., 2021). This condition is characterized by unpredictable and varying severity levels of relapse, causing patients to face an uncertain future (O’Mahony et al., 2019). In Israel, the annual incidence of POMS in 2021 was reported to be 1.5% among children, out of the total 5,000 cases of multiple sclerosis (MS) nationwide, and 4.5% among youth aged 14–18 (Israeli Ministry of Health, 2023).

Pediatric-onset multiple sclerosis often presents with multifocal symptoms, but as these children enter adolescence, the presenting symptoms are increasingly monofocal, resembling that of the adult form of the disease (Yamamoto et al., 2018). These symptoms include motor dysfunction (i.e., balance problems, muscle weakness, fatigue), visual and speech impairments, and cognitive dysfunction (i.e., attention, executive functions, and memory) (Storm van's Gravesande et al., 2019; Fisher et al., 2020). Additionally, the psychological consequences of POMS include feelings of incompetence and lower self-confidence, and social and physical anxiety, particularly when it occurs at a young age (Carroll et al., 2016; Payamani et al., 2016). As such, POMS has a pronounced negative effect on school-related activities (Carroll et al., 2016; Ghai et al., 2021) and can lead to a decreased quality of life (Storm van's Gravesande et al., 2019; Omranifard et al., 2023).

Given the above, POMS can present significant challenges and induce stress in the parents of affected children. They may experience feelings of worry and anxiety; heightened emotional, financial, and physical strains linked to both the immediate and long-term care of their children (Hebert et al., 2019; Maguire and Maguire, 2020); and a reduction in routine family activities (O’Mahony et al., 2019). Moreover, the complexity of the diagnosis process and difficulty maintaining a sense of continuity with their previously “normal” family life can further intensify parents’ sense of uncertainty and contribute to their distress (Hinton and Kirk, 2017; Cross et al., 2019). Indeed, a study by Uccelli et al. (2013) showed that parents of children with POMS (*n* = 15 couples) felt less satisfied with their parenting roles and exhibited a diminished sense of parenting competence than did couples with healthy children (*n* = 29).

The overall well-being of such parents is thus likely impacted, and although extensive research exists on how MS in parents affects the psychosocial and health conditions of children and adolescents (e.g., Andersen et al., 2023; Haker et al., 2023), very little research exists on how children’s MS affects parental well-being (Uccelli et al., 2013; O’Mahony et al., 2019, 2022). Furthermore, most of the studies that do exist are cross-sectional, despite the chronic nature of POMS and its long-term burdens, such as diminished daily functioning of the patient, financial strains, and the unpredictable progression of the disease, which are factors that could potentially diminish parental well-being (Hebert et al., 2019; O’Mahony et al., 2019). In this preliminary study we therefore explored two measures of well-being – namely, satisfaction with life and psychological distress – among Israeli mothers and fathers of youth diagnosed with POMS. We also examined changes over time, along three time-points, in these two measures of well-being.

Well-being represents the hedonic orientation according to which individuals achieve well-being by pursuing pleasure, enjoyment, and comfort, and avoiding discomfort (Huta and Ryan, 2010), and refers to “optimal psychological experience and functioning” (Deci and Ryan, 2008, p. 1). As stated, in the present study well-being was measured via satisfaction with life and psychological distress. *Satisfaction with life* can be viewed as a subjective cognitive assessment, or a statement regarding the quality of an individual’s life, which involves comparing one’s expectations and objectives against one’s ability to achieve them (Diener, 2008). *Psychological distress* refers to the unpleasant emotional state experienced in response to a specific stressor or demand, which results in temporary or permanent harm to the person (Ridner, 2004). Findings from the few studies that exist with regard to this measure (e.g., Hebert et al., 2019; Maguire and Maguire, 2020), including studies in the general area of MS (e.g., Giordano et al., 2016; Petrikis et al., 2019), indicate that caring for a person with MS is associated with lower levels of life satisfaction as well as higher depression and anxiety.

Hobfoll’s conservation of resources theory (Hobfoll, 1989) emphasizes the importance of resources in individuals’ ability to cope with stress, and – for the purposes of the current paper – a POMS diagnosis is certainly likely to be a stressor for parents of affected children. The fundamental concept of the conservation of resources theory is that individuals aim to acquire, maintain, and protect their valued material and psychosocial assets. Stress then arises from three scenarios: the actual depletion of resources, the potential risk of losing them, or the inability to obtain resources following a significant investment (Hobfoll, 1989, 2002). Based on Hobfoll’s theory, in this study we focused on perceived social support (PSS) – an interpersonal resource – and explored the association between PSS on the one hand and satisfaction with life and psychological distress on the other. *Perceived social support* encompasses an individual’s beliefs about the availability of various types of support from their social networks (Gottlieb and Bergen, 2010) and acts as a fundamental interpersonal resource, enhancing one’s capacity to navigate life’s challenges through personal, family, and social ties (Cohen et al., 2000). This support manifests in various ways: instrumental (e.g., financial help or assistance with daily tasks), informational (e.g., guidance or feedback on personal and familial matters), and emotional (expressions of empathy and caring) (Haber et al., 2007). Additionally, PSS is categorized on the basis of three distinct support sources: family (extended or nuclear), friends (individuals not related by family), and significant others (partners or those considered especially close) (Zimet et al., 1988). Researchers have linked PSS with mental and psychological health in general (Cohen and Wills, 1985; Szkody and McKinney, 2019) and with well-being and happiness in particular (Myers and Diener, 2018). Despite the limited research on satisfaction with life, psychological distress, and PSS in the specific context of POMS, a study by Bambara et al. (2014) did show that higher PSS levels correlated with fewer depressive symptoms in caregivers looking after veterans with MS, even when accounting for the severity of the disease and the veteran’s overall physical health.

Using a resource such as PSS may foster the utilization of coping strategies (Patterson, 2002). In this study, we focused on the strategy of “coping flexibility,” as proposed by Bonanno et al. (2011). Specifically, when confronting adversity or traumatic events, the authors argued that effective coping involves the flexible use of two coping processes. The first is termed *forward focus,* or the ability to distract oneself from a distressing/traumatic event, remain relaxed, ease distressed feelings, maintain a sense of humor, generate positive thoughts, retain one’s goals and plans, and be sensitive to others’ needs and well-being. The second is termed *trauma focus*, or the ability to avoid social interactions, focus on the distressing/traumatic event and appreciate its emotional and cognitive significance, generate realistic thoughts, and review and change one’s goals and plans. These two processes – forward focus and trauma focus – have been found to be essential for psychological adjustment to adversity/trauma (Bonanno et al., 2011; Rodin et al., 2017), and for simplicity’s sake, will henceforth be referred to as “coping flexibility.” Although there is limited research on post-traumatic stress symptoms associated with an MS or POMS diagnosis, in studies by Chalfant et al. (2004) and Counsell et al. (2013), post-traumatic stress symptoms were observed in individuals with MS, attributed to its potential life-threatening nature. In this preliminary study, coping flexibility was examined in a multilevel mediation model. Specifically, given that PSS is viewed as a resource for individuals to maintain or enhance their tangible and psychosocial resources, it could bolster parents’ ability to apply skills that facilitate their adjustment to this long-term disease (namely, their coping flexibility). This adjustment, in turn, might influence their well-being, reflected in elevated satisfaction with life and reduced psychological distress.

In the current study, we also focused on gender differences in the associations between PSS, coping flexibility, and well-being. Generally, in the context of MS, cross-sectional studies have shown that female caregivers tend to experience higher levels of distress, report more depressive symptoms, and perceive greater physical and emotional burdens than do male caregivers (Lee et al., 2015). Additionally, female caregivers have been found to express a greater need for emotional support than have their male counterparts (Lee et al., 2015). However, specific to POMS, there appears to be a lack of research regarding the impact of parents’ and patients’ gender on these variables.

## The current study

In the current preliminary study, using a longitudinal design, we explored the well-being of parents of children diagnosed with POMS, thus filling a lacuna in the literature. Specifically, we focused on changes in parental well-being at three time-points: 1 month, 6 months, and 12 months post-diagnosis. The study was grounded in Hobfoll’s conservation of resources theory (Hobfoll, 1989, 2002), and in it we examined a multilevel mediation model positing that the relationship between PSS and two outcomes — satisfaction with life and psychological distress — would be mediated by coping flexibility. Furthermore, we explored how these key variables’ trajectories over time varied according to the gender of both parents and patients.

## Methods

### Research design and participants

This preliminary longitudinal study was conducted at a tertiary university-affiliated children’s hospital in central Israel and was approved by the hospital’s institutional review board (IRB) (No. 0810-16-RMC). The third author, a social worker in the neurology department, initially contacted participants (i.e., parents) during their first visit to the neurology clinic. Parents were informed of the study’s purpose, eligibility criteria (i.e., being a parent of an adolescent diagnosed with POMS via clinical and MRI findings, and being fluent in Hebrew), and method (i.e., three assessments: 1 month, 6 months, and 12 months post-diagnosis). They provided their written consent and completed several standardized self-report questionnaires, which took approximately 15 min. From February 2018 through August 2021, 43 families of youth with POMS were contacted to participate in the study and asked to fill out the three self-report questionnaires at the three time-points. However, nine families declined to take part in the study due to a lack of interest in the study or a lack of time to fill out the questionnaires, and another ten families who initially joined the study provided incomplete data, which were thus unusable. As such, we obtained data from 36 parents (12 couples, *n* = 24, and 12 individual mothers and fathers): 14 fathers with a mean age of 51.07 (SD = 5.77) and 22 mothers with a mean age of 49.09 (SD = 5.90). Almost all of the participants (92%) were married; 56% (*n* = 20) had a bachelor’s degree and higher; and 47% (*n* = 17) described themselves as secular. Twenty-nine participants (81%) reported being employed. As for the youth diagnosed with POMS: 43% (*n* = 13) were boys with a mean age of 17.69 (SD = 2.93), and 57% (*n* = 23) were girls with a mean age of 17.87 (SD = 2.28). Children’s ages ranged from 10–18 years; all of them lived with their parents; and their expanded disability status scale (EDSS) score was at the lower end of the spectrum (EDSS 0–2.0).

Regarding the sample size, we conducted a power analysis to appraise the observed power to detect indirect effects (i.e., mediation effect, the central analysis in the current study). Using the Monte Carlo simulation (by employing the *power. Boot* function of the “bmem” R package), we found that the current sample allowed a power of 71.1% to detect an indirect effect of the size of *r* = 0.25 (6.25% explained variance).

### Measures

Participants completed standardized self-report questionnaires, all of which have been used previously in Israeli populations and have shown good psychometric properties.

*Sociodemographic characteristics* included details on participants’ gender, age, marital status, number of children, education, religion, and religiosity. In addition, information was obtained on patients’ gender, age, and school grade.

*Satisfaction with life* was measured using the 5-item Satisfaction with Life Scale (Diener et al., 1985). Participants evaluated statements such as, “In most ways my life is close to my ideal” on a Likert scale from 1 (*very strongly disagree*) to 7 (*very strongly agree*). High scores indicated high satisfaction with life. The original scale (Diener et al., 1985) had a Cronbach’s alpha of 0.84. The Hebrew adaptation of the satisfaction with life scale has been confirmed as both valid and reliable (Anaby et al., 2010). This measure was previously utilized to evaluate satisfaction with life among parents of children and youth diagnosed with neurological disorders (Sarwar et al., 2022). In the current study, Cronbach’s alphas were 0.83 at T1, 0.76 at T2, and 0.83 at T3.

*Psychological distress* was assessed by Kessler’s scale (K6; Kessler et al., 2002). This questionnaire provides a comprehensive measure of distress via six questions related to anxiety and depressive symptoms experienced in the last month, such as “During the last month, about how often did you feel restless or fidgety?” Items are rated from *none of the time* (1) to *all of the time* (5). A higher mean score indicates a higher level of psychological distress. The K6 has demonstrated good reliability, with Cronbach’s alpha values between 0.89 and 0.92 (Kessler et al., 2002). This measure was previously utilized to evaluate psychological distress among parents of children and youth diagnosed with neurological disorders (Diaz et al., 2021). The Hebrew adaptation (Ben-Ezra and Bibi, 2016) was shown to have a reliability of 0.88, and in our study, Cronbach’s alphas were 0.83 at T1, 0.74 at T2, and 0.86 at T3.

*Perceived social support* was measured by the Multidimensional Scale of Perceived Social Support (MSPSS; Zimet et al., 1990). The scale comprises 12 items, and a distinction is made between three PSS sources: family (4 items; e.g., “My family is willing to help me make decisions”); friends (4 items, e.g., “My friends really try to help me”); and a significant other (4 items, e.g., “There is a special person who is around when I am in need”). Participants answered on a 7-point Likert-type scale ranging from 1 (*very strongly disagree*) to 7 (*very strongly agree*). A mean score was calculated, with higher scores representing higher levels of PSS. The original MSPSS had an internal reliability of α = 0.91 (Zimet et al., 1988), and the Hebrew adaptation was shown to have a reliability of α = 0.91 (Ben-Ari and Gil, 2004). This measure was previously utilized to evaluate PSS among parents of children and youth diagnosed with neurological disorders (Hamama, 2022). In the current study, Cronbach’s alphas were as follows: for family support, 0.80 at T1, 0.89 at T2, and 0.85 at T3; for friends’ support, 0.80 at T1, 0.90 at T2, and 0.92 at T3; and for support from a significant other, 0.90 at T1, 0.85 at T2, and 0.87 at T3.

*Coping flexibility* was assessed by the Perceived Ability to Cope with Trauma Scale (PACT; Bonanno et al., 2011). The scale consists of 20 items that evaluate one’s perceived ability to engage in two coping strategies: (a) *forward focus*, with 12 items (e.g., “I remind myself that things will get better”); and (b) *trauma focus*, with eight items (e.g., “I let myself fully experience some of the painful emotions associated with the event”). Participants rate each item on a 7-point Likert scale from 1 (*not at all able*) to 7 (*extremely able*). A combined score is derived, representing the ability to utilize both coping strategies, where higher scores reflect a relatively greater ability to apply both. The Hebrew version’s Cronbach’s alphas were 0.91 for forward focus and 0.79 for trauma focus (Bonanno et al., 2011, p. 119). This measure was previously utilized to evaluate coping flexibility among parents of children diagnosed with neurological disorders (Hamama-Raz and Hamama, 2015). In the current study, we used a unified coping flexibility score indicating the ability to employ both coping processes (Bonanno et al., 2011). Cronbach’s alphas in the present study were 0.78 at T1, 0.88 at T2, and 0.94 at T3.

## Data analysis

The preliminary study comprised 36 participants: 12 couples (*n* = 24) and 12 individual mothers and fathers. These participants were observed over three measurement waves over a period of 12 months. Prior to the main analyses, we examined the normal distribution of all main study measures using a series of Shapiro–Wilk normality tests. We identified univariate outliers through the robust median absolute deviation (MAD) and multivariate outliers using the Minimum Covariance Determinant (MCD) method, with the latter being facilitated by the Routliers R package. Given that the three PSS measures showed significant negative skewness, the distress measures exhibited positive skewness, and both MAD and MCD analyses identified univariate and multivariate outliers, we employed robust analyses for all evaluations. In the first section of the results, we examined changes over time in the primary study measures – PSS, coping flexibility, satisfaction with life, and psychological distress. We also explored whether the trajectories of these changes varied on the basis of both the parents’ and the children’s gender. To achieve this goal, we utilized a series of robust mixed-effects models through the *rlmer* function of the *robustlmm* R package. The predictors were time (using months as the time unit: 0, 6, 12); parents’ and children’s gender (coded 0 = man/boy, 1 = woman/girl); and the interactions between gender and time with random slopes and intercepts based on couples’ identity and time. These models allowed us to examine linear changes over time in the study measures and to determine whether there were any differences in these changes between fathers/mothers or boys/girls (while taking into account the dependency between the fathers and mothers who were spouses). In the next section (Results), we delve into our primary theoretical framework—a multilevel causal mediation model. According to this model, PSS at T1 would predict the change in coping flexibility from T1 to T2, which in turn would predict the change in parents’ satisfaction with life and psychological distress from T1 to T3 (i.e., coping flexibility would act as a mediator between PSS, satisfaction with life, and psychological distress). To conduct this analysis, we employed a multilevel causal mediation model using the *mediate* function of the *mediation* R package. The significance of mediation paths was estimated using the quasi-Bayesian Monte Carlo method with 1,000 simulations.

## Results

### Pattern of associations between the study measures

**Figure 1:**
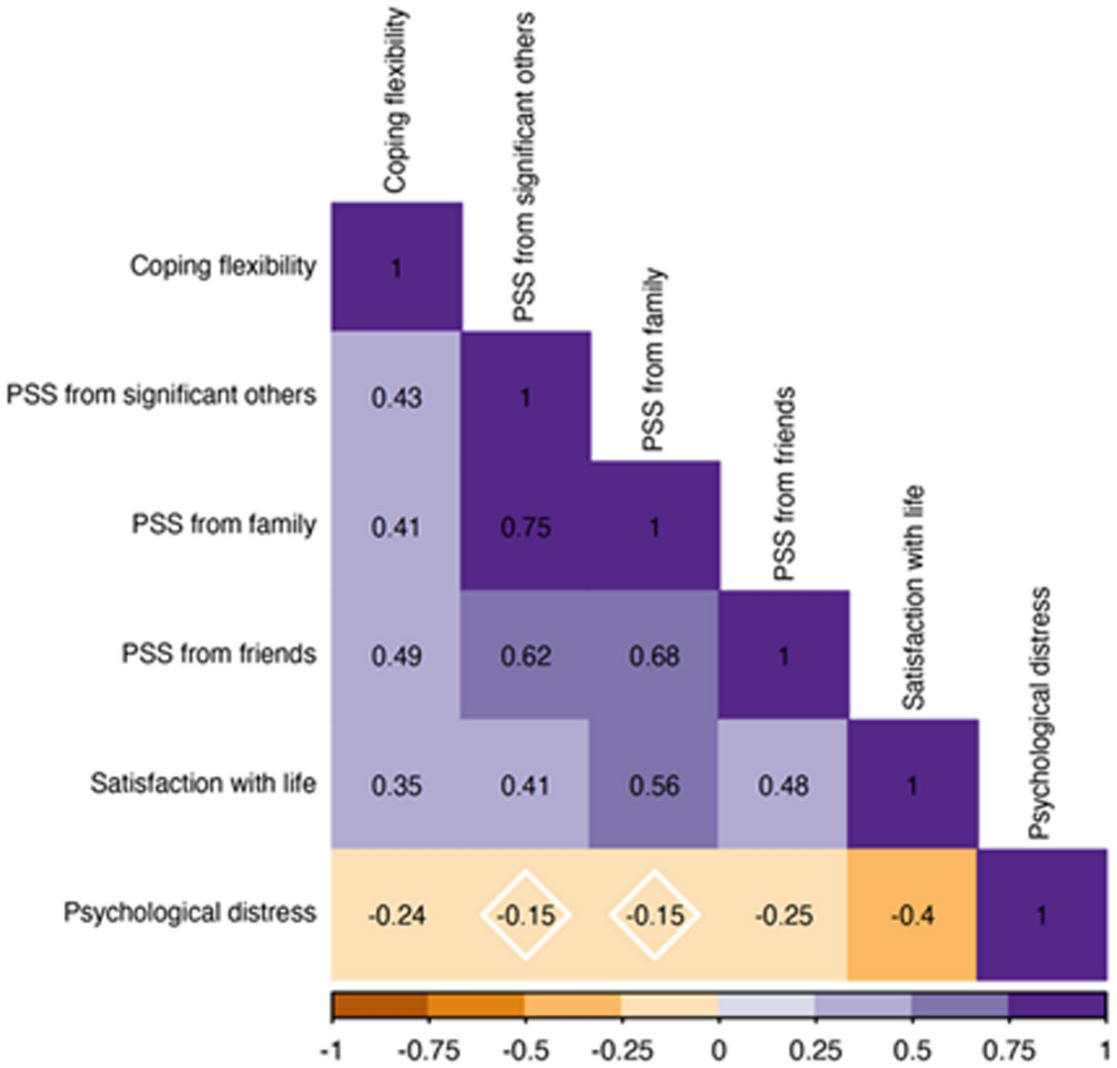
Pattern of associations between main study measures.

The pattern of associations between the main study measures (regardless of time) is presented in Figure 1. The analyses indicated that greater coping was linked with greater PSS, higher satisfaction with life, and lower psychological distress. Greater PSS was associated with higher satisfaction with life, although only support from friends (i.e., PSS from friends) was related to lower psychological distress. Finally, higher satisfaction with life was associated with lower psychological distress.

### Changes over time in the main study measures

Robust mixed-effects coefficients for the prediction of change are presented in Table 1. Means and standard deviations of all measures as a function of parents’ and children’s gender are presented in Table 2. The models indicated that over the course of the study (12 months), the reported levels of PSS, coping flexibility, satisfaction with life, and psychological distress remained unchanged among parents of youth with POMS. In addition, mothers and fathers who were married to each other, as well as individual mothers and fathers, of both boys and girls, tended to report similar levels over time. The sole significant difference was found in PSS from friends, such that mothers reported significantly greater support from friends than did fathers of youth with POMS (i.e., a main effect of parents’ gender on PSS from friends).

**Table 1:** Robust coefficients and their 95% confidence intervals for predicting changes over time in coping flexibility, perceived social support (PSS), satisfaction with life, and psychological distress as a function of parents’ and children’s gender.

Predictors	Coping flexibility			PSS from significant others			PSS from family		
	Estimates	CI	*p*	Estimates	CI	*p*	Estimates	CI	*p*
(Intercept)	99.78	88.80–110.76	<0.001	5.72	4.92–6.51	<0.001	5.68	4.92–6.44	<0.001
Time	0.33	−0.94 – 1.60	0.615	0.01	−0.07 – 0.09	0.814	0.00	−0.07 – 0.08	0.951
Parent’s gender (woman)	1.73	−5.46 – 8.91	0.638	0.07	−0.39 – 0.53	0.773	0.34	−0.09 – 0.76	0.122
Child’s gender (girl)	−5.12	−18.09 – 7.85	0.439	0.12	−0.83 – 1.08	0.803	−0.38	−1.30 – 0.53	0.413
Time × Parent’s gender	−0.47	−1.78 – 0.84	0.484	−0.05	−0.13 – 0.03	0.226	−0.05	−0.13 – 0.03	0.206
Time × Child’s gender	−0.90	−2.23 – 0.43	0.185	0.02	−0.06 – 0.11	0.581	0.03	−0.05 – 0.10	0.497
**Random effects**									
σ^2^	258.95			1.03			0.88		
τ_00_	168.56 _id_			1.01 _id_			0.94 _id_		
	0.00 _time_			0.00 _time_			0.00 _time_		
ICC	0.39			0.49			0.52		
N	24 _id_			24 _id_			24 _id_		
	3 _time_			3 _time_			3 _time_		
Observations	108			108			108		
Marginal *R*^2^ / Conditional *R*^2^	0.044 / 0.421			0.011 / 0.500			0.039 / 0.536		

Predictors	PSS from friends			Satisfaction with life			Psychological distress		
	Estimates	CI	*p*	Estimates	CI	*p*	Estimates	CI	*p*
(Intercept)	4.43	3.38–5.49	<0.001	4.99	4.20–5.79	<0.001	1.80	1.41–2.19	<0.001
Time	0.05	−0.04 – 0.13	0.280	0.02	−0.03 – 0.08	0.444	−0.03	−0.08 – 0.02	0.268
Parent’s gender (woman)	0.60	0.10–1.11	0.018	−0.25	−0.58 – 0.07	0.128	0.20	−0.08 – 0.48	0.167
Child’s gender (girl)	−0.12	−1.41 – 1.17	0.854	−0.23	−1.21 – 0.75	0.650	0.25	−0.20 – 0.71	0.276
Time × Parent’s gender	−0.06	−0.15 – 0.03	0.161	−0.00	−0.06 – 0.05	0.874	−0.01	−0.06 – 0.04	0.757
Time × Child’s gender	−0.03	−0.12 – 0.06	0.489	−0.04	−0.09 – 0.02	0.215	0.01	−0.04 – 0.07	0.610
**Random effects**									
σ^2^	1.19			0.49			0.42		
τ_00_	1.99 _id_			1.21 _id_			0.18 _id_		
	0.00 _time_			0.00 _time_			0.00 _time_		
ICC	0.63			0.71			0.30		
N	24 _id_			24 _id_			24 _id_		
	3 _time_			3 _time_			3 _time_		
Observations	108			108			108		
Marginal *R*^2^ / Conditional *R*^2^	0.037 / 0.639			0.022 / 0.718			0.068 / 0.350		

**Table 2:** Means and standard deviations of the main study measures as a function of parents’ and children’s gender.

Characteristic	Fathers, *N* = 14^a^	Mothers, *N* = 22^a^		Boys, *N* = 13^a^	Girls, *N* = 23^a^
Child’s gender			Parent’s gender		
Boys	6 (43%)	7 (32%)	Fathers	6 (46%)	8 (35%)
Girls	8 (57%)	15 (68%)	Mothers	7 (54%)	15 (65%)
Parent’s age	51.07 (5.77)	49.09 (5.90)		50.92 (5.12)	49.26 (6.25)
Child’s age	17.07 (2.97)	18.27 (2.07)		17.69 (2.93)	17.87 (2.28)
Coping flexibility at T1	98.64 (21.37)	102.55 (20.88)		100.38 (18.21)	101.39 (22.60)
Coping flexibility at T2	95.57 (19.48)	97.55 (18.54)		101.46 (20.96)	94.13 (17.14)
Coping flexibility at T3	95.14 (22.94)	94.32 (21.41)		102.31 (13.71)	90.30 (24.32)
PSS from significant others at T1	5.36 (1.74)	6.06 (1.35)		5.77 (1.63)	5.79 (1.50)
PSS from significant others at T2	5.75 (1.34)	5.73 (1.61)		5.73 (1.67)	5.74 (1.42)
PSS from significant others at T3	5.73 (1.09)	5.78 (1.31)		5.58 (1.34)	5.87 (1.16)
PSS from family at T1	5.12 (1.68)	5.90 (1.21)		5.94 (1.21)	5.40 (1.55)
PSS from family at T2	5.36 (1.55)	5.65 (1.46)		5.71 (1.38)	5.43 (1.56)
PSS from family at T3	5.38 (1.40)	5.47 (1.38)		5.63 (1.34)	5.32 (1.40)
PSS from friends at T1	4.07 (1.23)	5.10 (1.62)		4.71 (1.81)	4.70 (1.42)
PSS from friends at T2	4.25 (1.55)	5.03 (1.68)		4.77 (1.70)	4.71 (1.67)
PSS from friends at T3	4.34 (1.48)	4.69 (1.74)		4.85 (1.35)	4.39 (1.78)
Satisfaction with life at T1	4.59 (1.25)	4.54 (1.53)		4.62 (1.31)	4.52 (1.49)
Satisfaction with life at T2	4.79 (1.10)	4.58 (1.26)		5.03 (0.84)	4.45 (1.32)
Satisfaction with life at T3	4.67 (1.00)	4.57 (1.36)		4.86 (0.92)	4.47 (1.35)
Psychological distress at T1	2.18 (0.93)	2.36 (0.81)		2.33 (1.10)	2.26 (0.70)
Psychological distress at T2	2.05 (0.73)	2.16 (0.69)		1.94 (0.69)	2.22 (0.69)
Psychological distress at T3	1.87 (0.78)	2.04 (0.73)		1.86 (0.75)	2.03 (0.75)

a *n* (%); Mean (SD).

### Does coping flexibility mediate the effects of PSS on parents’ satisfaction with life and psychological distress?

**Figure 2:**
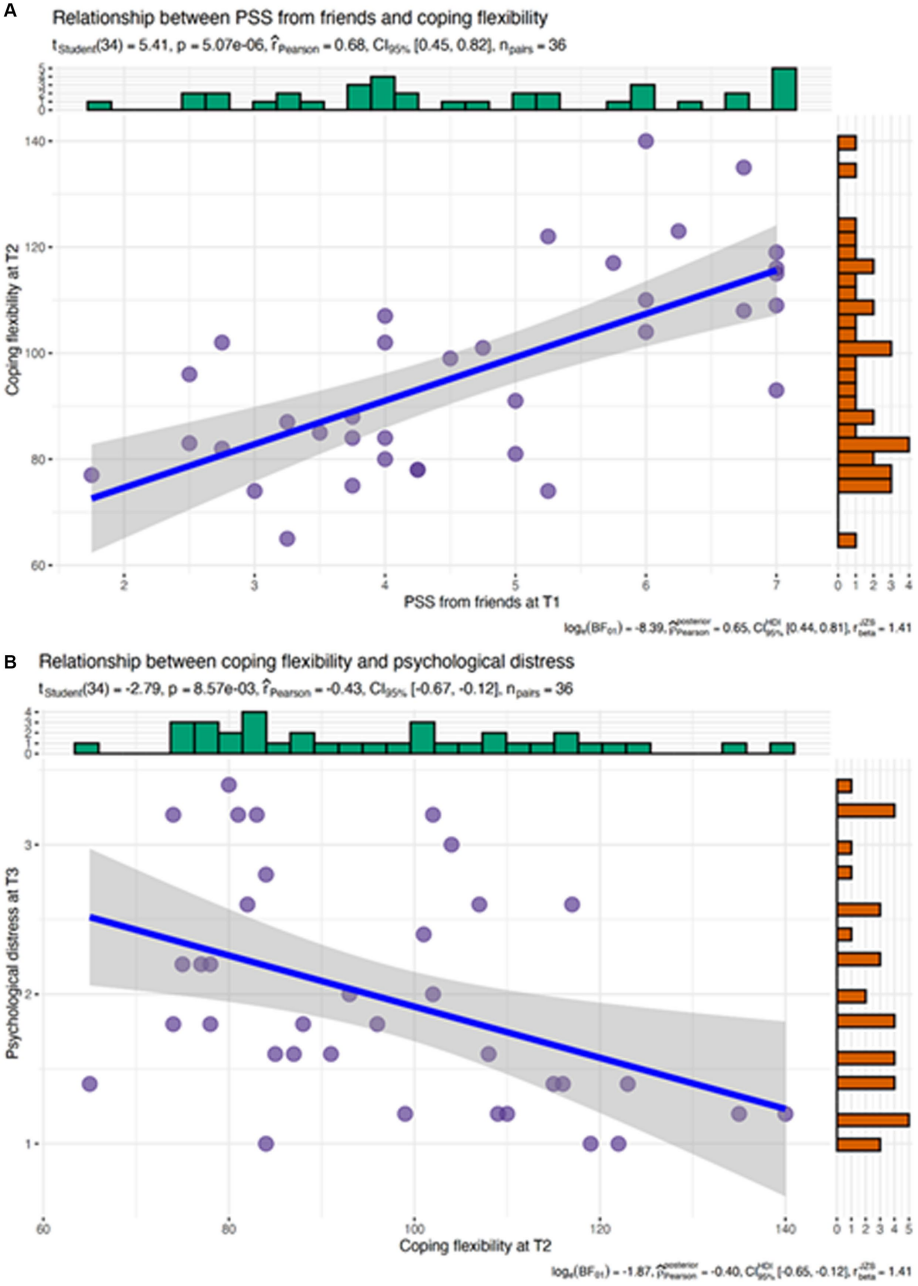
Pattern of the significant mediation path: PSS from friends at T1 significantly predicts coping flexibility at T2 **(A)**, which in turn predicts lower psychological distress at T3 **(B)**.

Results are presented in Table 3. The multilevel causal mediation model indicated that parents’ change in coping flexibility from T1 to T2 significantly mediated the effect of PSS from friends at T1 on the change in psychological distress from T1 to T3. Specifically, greater PSS from friends at T1 was significantly associated with an increase in coping flexibility from T1 to T2 (after controlling for the other sources of support). In turn, an increase in coping flexibility was associated with a decrease in psychological distress from T1 to T3 (after controlling for PSS; see Figure 2), *average causal mediation effects (ACME)* = −0.09, 95% *quasi-Bayesian confidence interval (CI_QB_)* -0.24, −0.01, *p* = 0.042. The causal mediation path via coping flexibility to satisfaction with life was not significant, *ACME* = 0.04, *CI_QB_* = −0.09, 0.20, *p* = 0.48.

**Table 3:** Multilevel causal mediation analysis.

Predictors	Coping flexibility at T2			Psychological distress at T3			Satisfaction with life at T3		
	Estimates	CI	*p*	Estimates	CI	*p*	Estimates	CI	*p*
Intercept	23.81	1.84–45.78	0.035	2.74	1.36–4.11	<0.001	1.20	−0.77 – 3.16	0.223
PSS from friends at T1	5.60	1.48–9.72	0.009	−0.16	−0.41 – 0.10	0.217	0.01	−0.37 – 0.38	0.975
PSS from family at T1	0.56	−3.52 – 4.64	0.781	0.04	−0.20 – 0.28	0.733	0.17	−0.14 – 0.48	0.263
PSS from significant others at T1	−0.59	−4.53 – 3.35	0.762	0.14	−0.09 – 0.36	0.218	−0.13	−0.43 – 0.17	0.396
Coping flexibility at T1	0.46	0.23–0.70	<0.001						
Coping flexibility at T2				−0.02	−0.03 – −0.00	0.049	0.01	−0.02 – 0.03	0.519
Psychological distress/Life satisfaction at T1				0.22	−0.06 – 0.49	0.116	0.53	0.27–0.79	<0.001
**Random effects**									
σ^2^	87.05			0.42			0.42		
τ_00_	50.78 _id_			0.00 _id_			0.51 _id_		
ICC	0.37						0.55		
*N*	24 _id_			24 _id_			24 _id_		
Observations	36			36			36		
Marginal *R*^2^ / Conditional *R*^2^	0.653 / 0.781			0.308 / 0.64			0.453 / 0.754		

In addition to the aforementioned findings, we further examined the primary research variables by comparing the current sample to a sample of Israeli parents of youth with neurodevelopmental disorders. These results are presented in Table 4. One-sample *t*-tests revealed that, when compared to Israeli mothers of children with autism spectrum disorder (ages 3–17; M = 10.98, SD = 4.28), parents in the current sample perceived significantly lower social support from friends. However, there were no differences in PSS from family members or significant others (Hamama, 2023). Conversely, parents in the current sample reported significantly higher levels of satisfaction with life than did Israeli mothers of children with neurodevelopmental disorders (ages 3–17; M = 12.08, SD = 3.39) (Hamama, 2022). Additionally, they reported significantly lower levels of psychological distress than did Australian parents of children with neurodevelopmental disorders (ages 2–17; M = 9.7, SD = 3.8) (Diaz et al., 2021).

**Table 4:** Comparison of the current sample with samples of parents of youth with other neurodevelopmental disorders.

	M	SD	M_Comparison_	*t*	*p*	Citation	Ethnicity
PPS from family members	18.39	5.77	18.17	0.23	0.821	Hamama (2023)	Israeli
PSS from friends	14.81	6.19	18.52	−3.60	<0.001	Hamama (2023)	Israeli
PSS from significant others	19.14	6.10	20.93	−1.76	0.087	Hamama (2023)	Israeli
Satisfaction with life	23.06	6.08	20.94	2.09	0.044	Hamama (2022)	Israeli
Psychological distress	5.88	4.55	13.92	−10.61	<0.001	Diaz et al. (2021)	Australian

## Discussion

To the best of our knowledge, the present preliminary longitudinal study is the first study in which Israeli parents of youth diagnosed with POMS have been examined in relation to PSS, coping flexibility, satisfaction with life, and psychological distress. We explored the changes that took place over the course of a year, addressing gender differences (mothers and fathers who were married to each other, as well as individual mothers and fathers, of both boys and girls). We discovered consistently stable reported levels of PSS, coping flexibility, satisfaction with life, and psychological distress throughout the year. Moreover, a sole significant difference was found between mothers and fathers in PSS from friends, such that mothers reported significantly greater support from friends than did fathers. No other differences were found between mothers and fathers who were married to each other, nor among individual mothers and fathers, of either boys or girls; participants tended to report similar levels over time. These results could potentially be attributed to the age of the participants’ children (i.e., mean age = 17.69 years, SD = 2.93). Specifically, adolescence is marked by changes in the parent–child relationship; namely, the previously “vertical” parent–child relationship, characterized by asymmetrical interactions, may shift to a “horizontal” one, characterized by equal, symmetrical, and reciprocal interactions (Branje, 2018). Hence, parental involvement in illness management might evoke more frequent and intense conflicts between parents and youth than those that might exist in parent-youth relationships where chronic illness is not a factor (Psihogios and Holmbeck, 2013). In this scenario, parents’ resources and well-being are continuously being challenged by both the developmental stage of the child and the POMS diagnosis, potentially explaining the consistency across time in the study’s main variables. Further, the small sample involved in the current study limited the ability to obtain a richer picture of possible change trends across time.

Regarding the significantly greater PSS from friends reported by mothers than fathers, a previous meta-analysis (Harandi et al., 2017) revealed similar findings: Women/ girls tend to discuss their emotional problems with friends more than men/boys do. This difference could be attributed to female social roles. Women/girls are often expected to be more adept at dealing with interpersonal issues than are men (Gaunt, 2013). Moreover, women tend to provide more emotional support and make better use of social support sources (Turner, 1994). Going forward, it would be worthwhile for researchers to explore the potential implications of gender differences and their impact on parental well-being – a matter of particular importance considering that variations in caring for patients with POMS could very well be linked to gender role expectations. Such variations are reflective of broader trends in the caregiving literature, indicating that women often assume the majority of caring responsibilities (Maguire and Maguire, 2020).

Interestingly, only PSS from friends was found to be associated with psychological distress, whereas PSS from all sources (family, friends, and/or significant others) was associated with satisfaction with life. Previous research has revealed that in facing difficult life events, support from friends predicts five of the six domains of psychological well-being (i.e., environmental mastery, positive relations with others, personal growth, purpose in life, and self-acceptance), whereas support from family predicts only two (i.e., positive relations with others and self-acceptance) (Secor et al., 2017).

Applying the conservation of resources theory (Hobfoll, 1989, 2002), we also explored a multilevel causal mediation model (i.e., coping flexibility would mediate the associations between PSS, satisfaction with life, and psychological distress). Our findings partially confirmed the model: Parents’ coping flexibility at T2 significantly mediated the association between PSS from friends at T1 and psychological distress at T3. However, the path to satisfaction with life was not significant.

Hobfoll’s conservation of resources theory (Hobfoll, 2002) identifies social support as a key resource that increases individual resilience and facilitates coping with life challenges. As such, it seems that parents who were equipped with PSS from friends at the time of diagnosis (T1) were able to activate coping flexibility at T2, which in turn seems to have led to lower psychological distress 12 months post-diagnosis (T3). Coping flexibility is not a uniform construct but an ongoing, multifaceted reaction to stressor variability (Bonanno et al., 2011). Hence, parents with better coping flexibility seemed able to deal more effectively with the emotional and physical burdens of caring for children with POMS, and thus reported lower psychological distress at T3. Another potential explanation can be found in Cohen and Wills’ (1985) direct effects hypothesis regarding the impact of social support on well-being. Namely, social support seems to have a direct positive effect on well-being, regardless of stress. As such, perhaps PSS from friends bolsters parents’ ability to cope with the demands of the child’s disease and prevents the situation from being appraised as highly stressful, and this appraisal is reflected in lower psychological distress.

Regarding the mediation model’s path to satisfaction with life, parents’ coping flexibility at T2 was not found to mediate between PSS from friends at T1 and satisfaction with life at T3. Ali et al. (2010) differentiated between life domain satisfaction (i.e., satisfaction with specific areas of an individual’s life such as work, marriage, and income) and overall satisfaction with life (a broad, comprehensive judgment of life). As such, coping flexibility might not have played a mediating role given that parents’ judgment of their life, overall, is likely much broader than just their parenting of a child with a long-term disease.

Several limitations should be noted. First, this study was conducted at one site, included a small number of respondents, had a high attrition rate (approximately 46%), and had no control group. Additionally, the unique sociocultural environment and healthcare system of Israel might not accurately reflect the experiences of parents of children with POMS in different regions or countries. Consequently, these findings should be viewed as preliminary, and any generalizations should be made with caution; in the future, studies should be conducted to include a broader population and control group. Second, the study’s variables were measured by self-report questionnaires and thus were subject to bias including social desirability and recall biases. Future studies should include structured interviews to provide additional information and to help researchers obtain a comprehensive understanding of those factors that might reduce parents’ well-being. Third, as there wasn’t sufficient variability in the children’s disability status (the EDSS score was at the lower end of the spectrum), future researchers might consider examining the role of children’s disability status (EDSS) in relation to parents’ well-being. Lastly, T2 and T3 data were obtained during the COVID-19 pandemic: a stressor triggering psychological distress among parents in general, regardless of whether their children did or did not have a chronic condition (van Tilburg et al., 2020). As such, we were unable to ascertain whether PSS, coping flexibility, and psychological distress were pre-existing, exacerbated, or acutely caused by the POMS diagnosis. Researchers should establish the mediating path and clarify the role of coping flexibility vis-à-vis parents’ well-being. Further, information gathered about the effects of POMS on everyday life and the parent-youth relationship might help explain the variability over time in parents’ satisfaction with life and psychological distress.

Despite these limitations, the present preliminary findings provide new insights into the role of different PSS sources. Specifically, PSS from friends was found to be associated with less psychological distress, and greater PSS from friends was found to be significantly associated with better coping flexibility, which was in turn associated with lower psychological distress. Thus, researchers should utilize the three components of the PSS measure (family, friends, and significant others) and not only the total PSS score, to provide richer perspectives regarding the outcome variables. Additionally, given the scant research on parents of youth with POMS, the current study contributes to the literature by elaborating on resources (i.e., PSS and coping flexibility) that may be associated with parents’ well-being. The findings should heighten clinicians’ awareness of the personal and interpersonal resources that may be associated with well-being in the context of parenting children with long-term illnesses. Future research based on a larger sample, examining the pattern of associations across time that emerged from this exploratory longitudinal study, could lead to greater psychosocial support for parents of youth with POMS. Finally, our findings emphasize the notion that professionals should acknowledge the meaningful role played by PSS from friends in lowering mothers’ psychological distress, as these findings could be applicable in terms of working with fathers as well. Interventions aimed to promote support from parents’ social/family networks in managing caregiving stress and improving parents’ well-being should be enhanced. We thus encourage healthcare professionals to identify the specific needs of families in terms of psychological, social, educational, informational, and financial support. Families should be informed about resources available within the community, and psychological interventions are recommended to be tailored with sensitivity to gender-specific needs.

## Data availability statement

The raw data supporting the conclusions of this article will be made available by the authors, without undue reservation.

## Ethics statement

The studies involving humans were approved by Schneider Children’s Medical Center, Petach Tikva, Israel; 0810-16-RMC. The studies were conducted in accordance with the local legislation and institutional requirements. The participants provided their written informed consent to participate in this study.

## Author contributions

LH: Conceptualization, Formal analysis, Investigation, Methodology, Writing – original draft, Writing – review & editing. YH-R: Conceptualization, Formal analysis, Investigation, Methodology, Writing – original draft, Writing – review & editing. KL-S: Data curation, Investigation, Writing – review & editing. EG-C: Conceptualization, Data curation, Investigation, Writing – review & editing.
